# Angiolipomatous Atypical Presentation of Dercum’s Disease

**DOI:** 10.7759/cureus.105131

**Published:** 2026-03-12

**Authors:** Mariko A Locke, Nicholas S Tedesco

**Affiliations:** 1 College of Osteopathic Medicine of the Pacific-Northwest, Western University of Health Sciences, Lebanon, USA; 2 Orthopedic Surgery, Good Samaritan Regional Medical Center, Corvallis, USA; 3 Orthopedic Oncology, Good Samaritan Regional Medical Center, Corvallis, USA

**Keywords:** adiposis dolorosa, angiolipomas, dercum’s disease, extremity soft tissue tumor, painful lipomatosis

## Abstract

We describe the case of a 35-year-old man presenting with multiple painful soft tissue masses in bilateral upper extremities, the trunk, and groin. The patient had a prior history of painful lipomatosis in the left upper extremity with multiple masses removed without intraoperative or postoperative complications. Physical examination revealed multiple right upper extremity nodules that triggered reproducible pain upon palpation. Magnetic resonance imaging (MRI) confirmed the presence of multiple subcutaneous lipomatous masses with variable nonlipomatous internal signal in some of the masses. He was managed surgically, and 36 distinct subcutaneous soft tissue masses were resected across three operations. Histopathology following these resections revealed 13 angiolipomas, a rare finding for Dercum’s disease, and 23 lipomas.

This is the first reported case of Dercum’s disease presenting with multiple angiolipomas in the absence of prior trauma to the affected region, distributed across multiple body regions rather than localized to one specific extremity.

## Introduction

Dercum’s disease (adiposis dolorosa) is a rare disease that most commonly affects adult women and is characterized by numerous, painful subcutaneous neoplastic adipose tissue nodules, known as lipomas [1]. Common sites for lipomas are the legs, arms, trunk, buttocks, and thighs [2,3]. Dercum’s disease is associated with chronic pain (longer than three months) in adipose tissue, obesity or metabolic syndrome, sleep disorders, and neurological or psychiatric disturbances [2,3]. The prevalence of this disease remains unknown, likely because this disease is rare and lacks standardized diagnostic criteria [3]. Most cases are sporadic, though some evidence suggests a genetic component that is potentially inherited in an autosomal dominant manner with variable expressivity [4]. However, no specific mutation has been identified for this disease [3].

The underlying pathology of this disease is poorly understood, but at least one study suggests an inflammatory mechanism [5]. A biopsy of a lipoma from a previously reported case of a 54-year-old female patient with Dercum’s disease revealed intralesional inflammation, supporting the inflammatory mechanism theory [6]. In contrast, previous studies have reported normal or slightly elevated ESR and CRP levels in most cases of Dercum’s disease, suggesting that inflammation is not the only mechanism underlying the pathology [3]. Another recent study noted distinct immunohistochemistry markings, such as increased leukocytes, platelets, and basophils, while observing a lower number of natural killer cells [5]. Other potential mechanisms proposed are trauma-induced lipoma formation or lymphatic dysfunction [2,3].

We present an unusual case of Dercum’s disease in a 35-year-old man with multiple painful lipomas and angiolipomas affecting the bilateral upper extremities and the trunk. This case demonstrates the importance of ongoing research regarding the pathophysiology of Dercum’s disease and the possibility that it exists as a spectrum of disease.

## Case presentation

**Figure 1:**
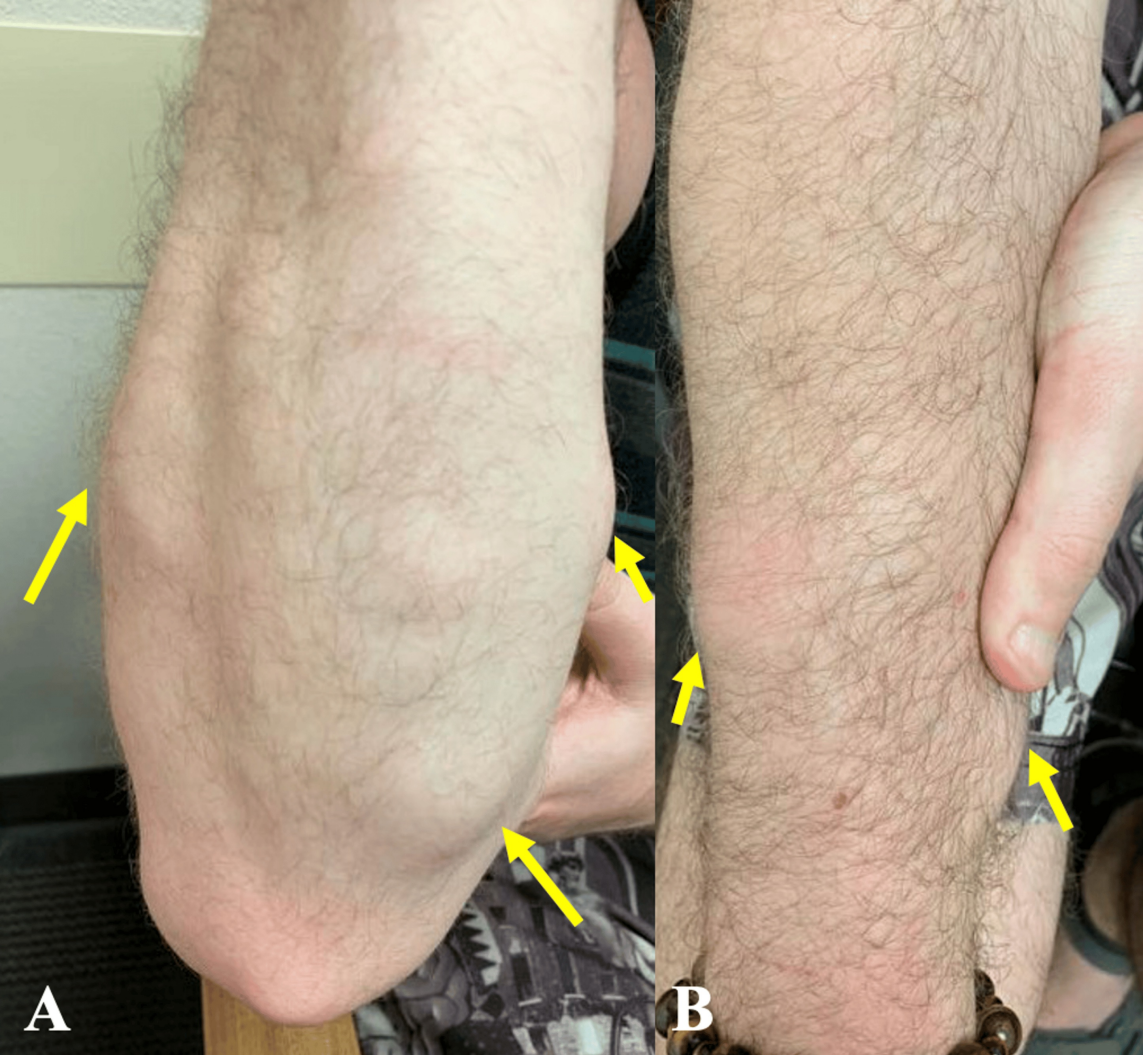
Multiple views of subcutaneous soft tissue masses located near the right elbow. Two views showing multiple subcutaneous soft tissue enlargements (yellow arrows) of various sizes distributed next to the right elbow (A) and throughout the dorsal antebrachium (B). Sizes of the masses in the right upper extremity ranged from 1.5 to 3.9 cm in longest diameter.

A 35-year-old man presented to an orthopedic oncology clinic with multiple, painful subcutaneous nodules on the right forearm and a painful right elbow mass with associated numbness and tingling in an ulnar distribution with direct contact. Per the patient, these painful nodules had been present for three years prior to presentation. The nodules were not painful at rest, though he reported up to 10 out of 10 pain with direct contact. The patient had a history of multiple, painful lipomas on his back and left upper extremity, treated by surgical removal. No post-operative complications were reported following previous surgeries, and he had no history of previous trauma in any regions of tumor presence. His BMI was 23.0 at this presentation. On physical exam, multiple soft, mobile, subcutaneous, tender masses surrounded the right ulnar nerve at the elbow, medial distal brachium, and multiple masses on the dorsal forearm (Figure 1). Palpation reproduced the patient’s symptoms. The patient reported decreased subjective sensation to light touch in the right ulnar nerve distribution.

**Figure 2:**
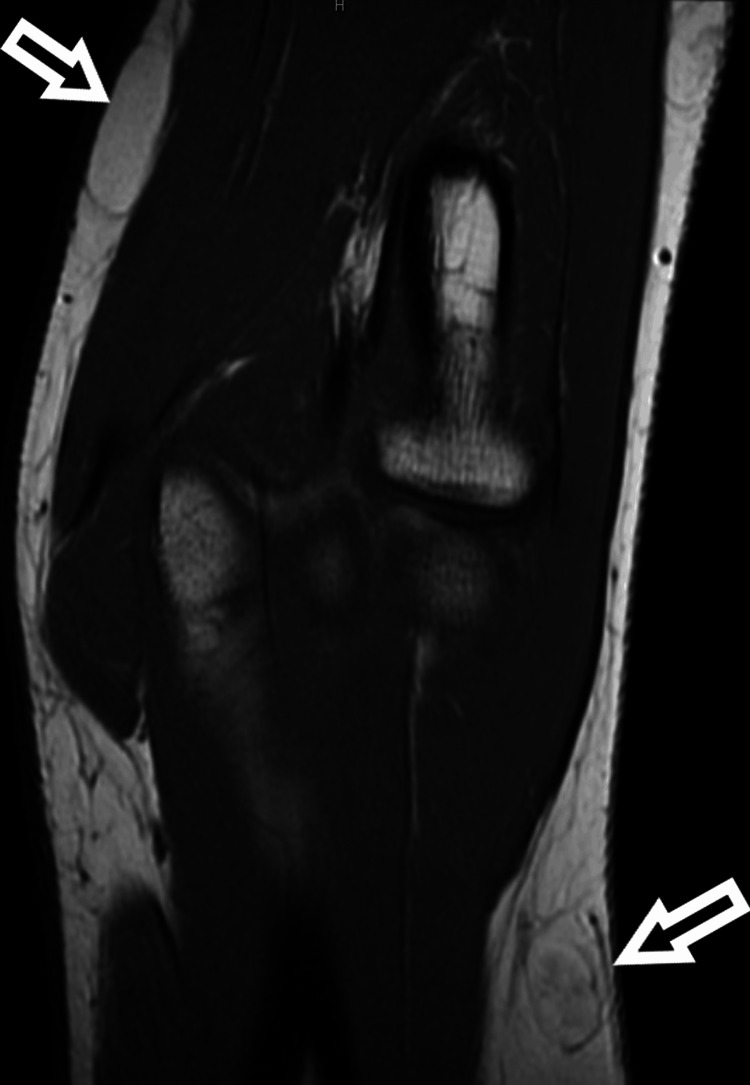
Sagittal T1-weighted MRI of the right elbow demonstrating multiple soft tissue masses in the brachial and antebrachial regions with varying degrees of non-adipose tissue.

**Figure 3:**
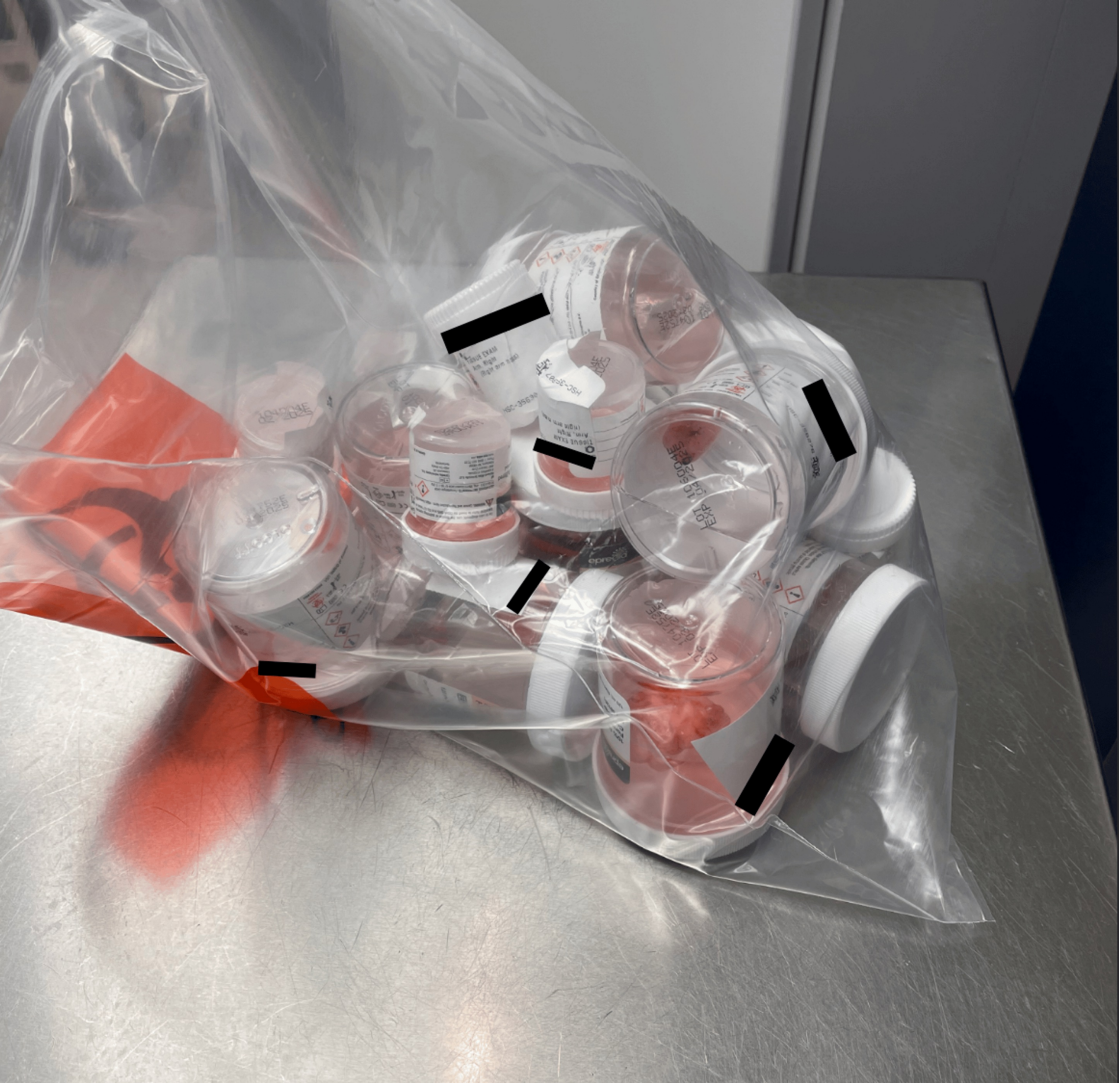
A total of 17 distinct masses of variable sizes were surgically removed along the right brachium and antebrachium following the first operation.

**Figure 4:**
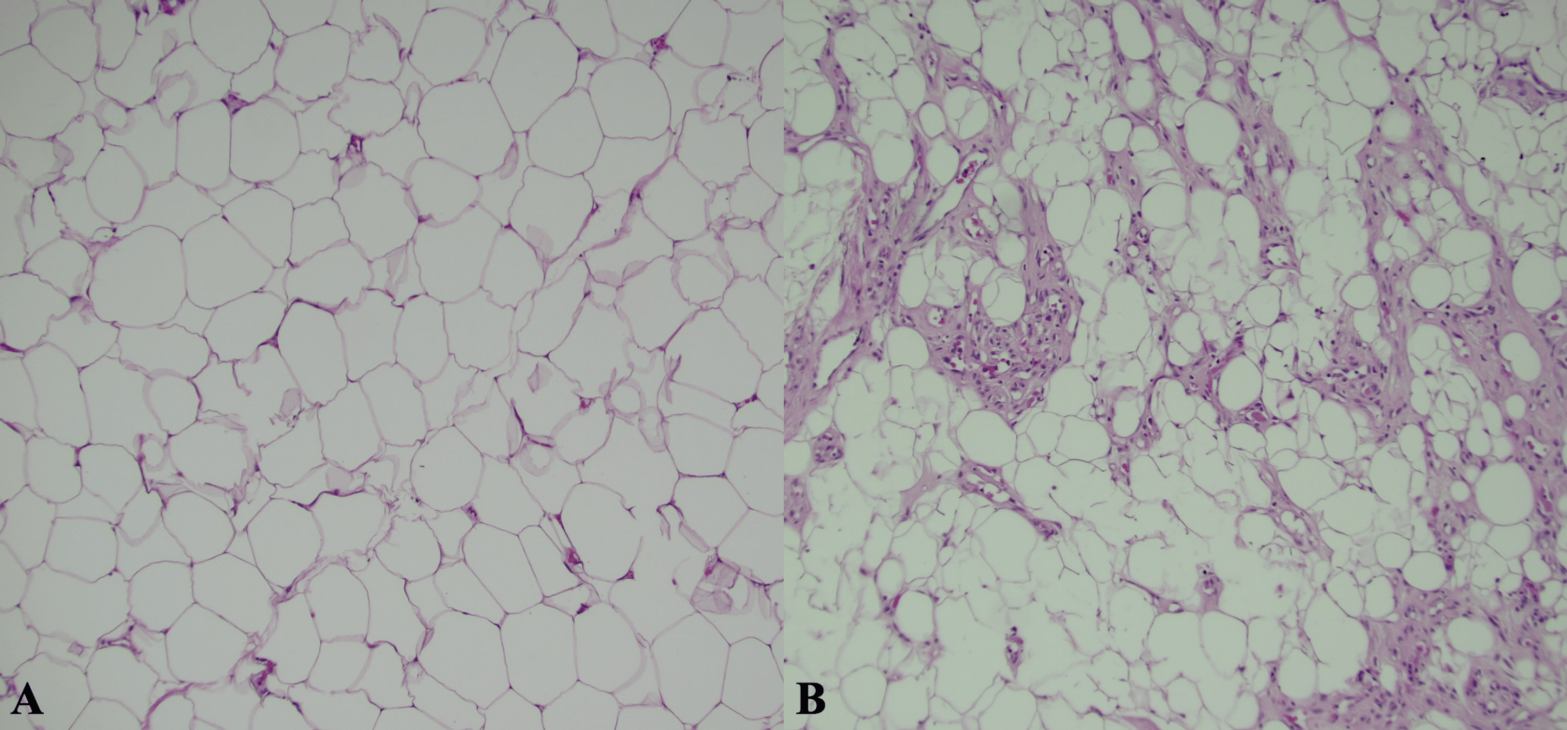
Histological sections from the first operation of the right upper extremity. Two 100x photomicrograph histological sections of masses from the right brachium and antebrachium. Note the mature adipocyte deposition with minimal vascularity, characteristic of a lipoma (A). Note the mature adipocytes with increased vascularization, characteristic of an angiolipoma (B).

Magnetic resonance imaging (MRI) of the right elbow revealed multiple small, subcutaneous lipomas (Figure 2), some with intralesional stranding and nonfatty tissue. The diagnosis of Dercum’s disease was consistent with the patient’s clinical picture and known history of painful lipomatosis. The patient was offered surgical resection of the masses from the right brachium and antebrachium. Informed consent was obtained. Intraoperatively, 17 distinct masses were removed (Figure 3). Pathology revealed six lipomas and 11 angiolipomas (Figure 4). Postoperatively, the patient reported improvement in his pain and nerve symptoms in the right upper extremity. Physical exam revealed no evidence of erythema, infection, wound dehiscence, or palpable masses within surgical beds. The patient had a full range of motion in the right elbow and shoulder.

Once fully recovered from the right upper extremity resections, the patient reported additional pain in multiple soft tissue masses along the left trunk and groin. Physical exam revealed several tender subcutaneous masses in the left lower quadrant of the abdomen along the inferior rib margin, left dorsal lumbar trunk, and left groin without evidence of bowel herniation. The patient opted for surgical resection of the soft tissue masses, of which 10 distinct masses were excised intraoperatively with histopathology revealing all lipomas. Postoperatively, the incision sites demonstrated no signs of infection or wound dehiscence. The patient reported improvement in symptoms.

During follow-up, the patient again reported recurrent pain in multiple additional subcutaneous masses localized to the left abdomen, as well as a combination of new lesions in bilateral arms and recurrent lesions surrounding the left elbow that were previously removed by another provider. Given the patient’s success with prior excisions, the patient decided to proceed with another excision in which nine masses were resected. Pathology later revealed that four lipomas were removed from the left abdomen, three lipomas and one angiolipoma from the left arm, and one angiolipoma from the right arm. Postoperatively, the patient reported improvement in pain and a return to daily activities. The last visit occurred six and a half weeks after the final operation, which was one and a half years following the first surgery, where he was released without complications.

## Discussion

This case illustrates an atypical presentation of Dercum’s disease in a non-obese man with multiple painful angiolipomas combined with multiple painful lipomas in multiple body regions. Previous literature has reported middle-aged, obese females presenting with painful subcutaneous lipomatosis [1,2]. Given the patient’s demographics and rarity of the histologic subtype of lipoma, this may represent a previously unreported, distinct disease or suggests that Dercum’s disease lies on a spectrum not previously recognized.

This presentation is similar to familial multiple angiolipomatosis, as multiple angiolipomas present on the upper extremities in younger males [7]. This is less likely as the patient’s family history was not notable for painful masses or similar lesions. Familial multiple lipomatosis (FML) could be considered as well; however, the hallmark feature of FML is painless lipomatosis [5]. In contrast, Dercum’s disease is characterized by painful adipose tissue and was also present in this patient [5]. Given the presence of multiple painful angiolipomas and lipomas, it is possible this patient may have Dercum's disease and another concomitant lipomatosis, such as angiolipomatosis, though this would be exceedingly rare, as these conditions in isolation are rare and lack a known prevalence [3]. Additionally, his interval development of pain in previously painless masses leading to multiple, progressive surgeries is also a unique and poorly understood variable of this case.

Angiolipomas are a variant of lipomas, characterized by the presence of vessels and adipocytes [5]. Previous literature suggests formation in post-traumatic settings or response to hormones [5]. A total of 13 angiolipomas were removed from the patient’s upper extremities and trunk. Angiolipomas are not typically associated with Dercum’s disease, with few case reports mentioning the presence of angiolipomas [8]. Hao et al. reported angiolipoma and angiomyolipoma formation in the region of a previous traumatic injury in a 39-year-old obese male patient with a history of previous angiolipoma removal [8]. He was treated with a transcutaneous frequency rhythmic electrical modulation system (FREMS) and multiple pain medications, reporting decreased pain [8]. In contrast, our patient had no history of trauma to the affected regions and was managed surgically. Though these cases are similar, they suggest that angiolipoma development in Dercum’s disease may not be related to trauma.

There is no universal standard of care for the management of Dercum’s disease. Treatment is often aimed at symptom relief and depends on the patient’s symptoms [2,5]. In this case, the patient decided to proceed with surgery due to the size, location, and number of the lesions affecting the patient’s quality of life. Surgical removal of the masses may provide targeted relief when masses impinge on surrounding structures or cause functional impairment [2]. It remains a reasonable option in cases where pain is refractory to conservative management. However, recurrence of lesions after surgical removal has been reported and was observed in this patient on his left upper extremity, which was treated several years prior by another provider [3,5]. Non-operative management includes analgesics such as non-steroidal anti-inflammatory drugs, lidocaine, systemic corticosteroids, and FREMS [3,4,9,10].

## Conclusions

In summary, this case highlights a widespread distribution of multiple painful angiolipomatosis and lipomatosis in the absence of trauma or other predisposing conditions in a young male with Dercum’s disease. It remains unclear whether trauma may trigger an angiolipomatous response in susceptible individuals or whether there is a genetic link between angiolipomatosis and painful lipomatosis. As this disease is rare and difficult to study, further case reports and investigations are vital to determine whether Dercum’s disease encompasses a spectrum and to reveal the underlying pathophysiology of this poorly understood condition.

## References

[1] Charifa A, Azmat CE, Badri T (2025). Lipoma pathology. StatPearls [Internet].

[2] Kucharz EJ, Kopeć-Mędrek M, Kramza J, Chrzanowska M, Kotyla P (2019). Dercum's disease (adiposis dolorosa): a review of clinical presentation and management. Reumatologia.

[3] Hansson E, Svensson H, Brorson H (2012). Review of Dercum's disease and proposal of diagnostic criteria, diagnostic methods, classification and management. Orphanet J Rare Dis.

[4] Campen R, Mankin H, Louis DN, Hirano M, Maccollin M (2001). Familial occurrence of adiposis dolorosa. J Am Acad Dermatol.

[5] Dupuis H, Lemaitre M, Jannin A, Douillard C, Espiard S, Vantyghem MC (2024). Lipomatoses. Ann Endocrinol (Paris).

[6] Baig MA (2023). An unusual presentation of Dercum's disease to the emergency department. Oxf Med Case Reports.

[7] Garib G, Siegal GP, Andea AA (2015). Autosomal-dominant familial angiolipomatosis. Cutis.

[8] Hao D, Olugbodi A, Udechukwu N, Donato AA (2018). Trauma-induced adiposis dolorosa (Dercum's disease). BMJ Case Rep.

[9] Desai MJ, Siriki R, Wang D (2008). Treatment of pain in Dercum's disease with Lidoderm (lidocaine 5% patch): a case report. Pain Med.

[10] Caretto A, Errichiello E, Patricelli MG (2021). Transcutaneous electrical stimulation therapy and genetic analysis in Dercum's disease: a pilot study. Medicine (Baltimore).

